# A Rare Case of Malignant Infantile Osteopetrosis Presenting as Frontal Lobe Hemorrhage

**DOI:** 10.7759/cureus.9234

**Published:** 2020-07-17

**Authors:** Aiman Ali, Taha Bin Arif, Sulhera Khan, Fasiha Bakhtawar Fatima, Rabia Sehar Abbasi

**Affiliations:** 1 Internal Medicine, Dow University of Health Sciences, Karachi, PAK; 2 Medical Education and Simulation, Dow University of Health Sciences, Karachi, PAK; 3 Internal Medicine, Civil Hospital Karachi, Dow University of Health Sciences, Karachi, PAK; 4 General Surgery, Liaquat University of Medical and Health Sciences, Hyderabad, PAK

**Keywords:** malignant infantile osteopetrosis, genetic, convulsions, hemorrhage, hypocalcemia, hematopoietic stem cell transplant, pancytopenia

## Abstract

Osteopetrosis comprises a group of rare inherited disorders of the bones characterized by a common radiographic finding of increased bone thickness. The disorders vary genetically as well as clinically, and range in severity from mild manifestations to fatal complications based on the type of the disorder. Malignant infantile osteopetrosis (MIOP) is a less common, more severe form of the disease with most affected individuals surviving up to only the first few years of life if left untreated. We present a previously diagnosed case of the malignant infantile type who was brought to our attention for convulsions. Antiepileptic medications were started along with supportive therapy. CT scan revealed a large frontal lobe hemorrhage, which was suspected as a possible cause of the seizures, other than the progressively worsening hypocalcemia. Laboratory investigations also revealed pancytopenia and blood cultures positive for staphylococci, which were treated accordingly. Genetic testing and hematopoietic stem cell transplantation could not be performed due to financial constraints and the rapidly deteriorating condition of the patient. Unfortunately, the baby expired two weeks from the day of admission. This case highlights a rare and grave clinical manifestation of MIOP and brings to attention the significance of bone marrow transplantation as the only curative therapy of the disease.

## Introduction

Osteopetrosis, also known as marble bone disease or Albers-Schonberg disease, refers to a group of exceedingly rare genetic skeletal disorders characterized by a radiographic appearance of excessive bone growth, density, and thickness. The disorder is caused by a defect in bone resorption and remodeling due to differentiation or functional defects in osteoclasts [1]. Osteopetrosis is broadly categorized into two forms: autosomal-dominant osteopetrosis (ADO) and autosomal-recessive osteopetrosis (ARO). ADO has an incidence of about one in 20,000 births. It is usually diagnosed incidentally and remains asymptomatic or may show mild symptoms in late childhood or adult life [1]. The latter, also called malignant infantile osteopetrosis (MIOP), is the rarer, more severe form that may even prove to be fatal if left untreated. It has an average incidence of 1: 200,000-1: 300,000 live births and is diagnosed in utero or within the first few years of life [2].

The clinical manifestations may vary, ranging from mild features in the adult type or life-threatening complications in the infantile type [1]. MIOP is characterized by macrocephaly with frontal bossing and a broad face, enamel hypoplasia, bone pain with multiple fractures, and tetanic seizures secondary to hyperparathyroidism and hypocalcemia. Children also present with stunted growth and failure to thrive. Excessive skull growth and narrowed cranial foramina result in impingement of cranial nerves, most commonly optic, auditory, and facial nerves, which may lead to blindness, hearing loss, and facial paralysis, respectively. Pancytopenia, recurrent infections, and hepatosplenomegaly occur as a result of bone marrow failure and extramedullary hematopoiesis [3].

The diagnosis is made based on clinical presentation, radiographic findings, and gene analysis. Treatment is mainly symptomatic and supportive and requires a multidisciplinary team. Bone marrow transplantation of hematopoietic stem cells is the only definitive treatment of MIOP, which has a poor prognosis as compared to the benign adult form, which has better outcomes and a normal life expectancy [4,5]. Here we report a rare case of a three-year-old male child diagnosed with MIOP who presented with generalized seizures.

## Case presentation

A three-year-old male child, vaccinated to date, weighing 8 kg, known case of MIOP diagnosed two months back presented to the pediatric emergency department (PED) at Civil Hospital Karachi, with complaints of active convulsions. The seizures were afebrile, generalized tonic-clonic in nature, and were accompanied by nystagmus, facial twitching, and abnormal movements. The episode was not associated with vomiting, drooling, and urinary or fecal incontinence, and lasted for about 20 minutes. History was also negative for convulsions. The child was the first progeny of consanguineous marriage and was delivered at term via normal vaginal delivery at home. There was no family history of miscarriage, stillbirth, any chronic illness, or genetic diseases. Developmental milestones were achieved typically by one year of age but started to regress after that. Later, the gross and fine motor development halted, and a progressive decline in social and cognitive growth was seen.

History dates back to infancy when the child developed repeated chest infections and had multiple hospital admissions. He was diagnosed with pneumonia and was managed accordingly. Two months ago, the child presented with complaints of generalized bone pains, pallor, and abdominal distention. On examination, hepatosplenomegaly was identified. Laboratory investigations indicated pancytopenia and hypocalcemia. Based on clinical, laboratory, and radiological findings, a definitive diagnosis of osteopetrosis was made. The patient was managed for bone pains and was transfused packed red cells for anemia. The family was counseled about the disease and its feared complications. Bone marrow transplant was suggested as the definitive cure but unfortunately could not be performed due to financial constraints.

General physical examination of the patient concluded a pale-looking, malnourished, stunted child with widespread petechiae and bruises all over the body. The child was afebrile with a heart rate (HR) of 120 beats/min, blood pressure (BP) of 115/66 mmHg, respiratory rate (RR) of 40 breaths/min, and oxygen saturation (SaO_2_) of 98% at room air. Head and neck examination showed palpable submandibular lymph nodes, frontal bossing, flattened malar eminence, and deviation of the mouth to the left side. Oropharyngeal examination revealed malformed teeth with dental caries. Findings suggestive of malnutrition, such as the rachitic rosary and bow legs, were also seen. On abdominal examination, a distended, tense abdomen with prominent veins was found. The liver could not be palpated due to the abdominal distention, while an enlarged spleen measuring 17 cm was identified on palpation. Central nervous system examination revealed normal bulk, decreased power, hyperreflexia, and hypertonia in all four limbs with a low Glasgow Coma Scale (GCS) score of 9. The rest of the examinations were insignificant.

The initial laboratory investigations (at the day of admission) revealed low hemoglobin (Hb) of 8.6 g/dL, decreased hematocrit of 27%, severely decreased total leukocyte count (TLC) of 1.2 x 10^9^/L, and a low platelet count (PLT) of only 22 x 10^9^/L. The peripheral smear revealed normochromic normocytic anemia, anisocytosis, leukopenia, and multiple platelet clumps. Biochemical investigations revealed hypocalcemia (Ca = 5.6 mg/dL) and slightly decreased phosphate levels. Renal function tests, coagulation profile, and urinalysis were within normal ranges (Table 1). Blood culture was positive for coagulase-positive staphylococci sensitive to vancomycin. The patient was kept nil per oral (NPO), and regular monitoring of his vitals was carried out. Intravenous meropenem, vancomycin, and acyclovir were administered eight hourly. Electroencephalogram (EEG) showed polyspike-wave pattern suggestive of generalized tonic-clonic seizures. Phenytoin and sodium valproate were commenced for the seizures, while active seizures were managed with diazepam. Platelets and packed red blood cells were transfused. Calcium gluconate diluted in 10% dextrose water was administered over 30 minutes to reverse the hypocalcemia. An initial rise in serum calcium level was seen, which was followed by decreasing levels throughout the stay (Table 1).

**Table 1:** Laboratory investigations over the course of hospital stay. Hb, hemoglobin; Hct, hematocrit; TLC, total leukocyte count; PLT, platelets; CRP, C-reactive protein; BUN, blood urea nitrogen; Cr, creatinine; Na, sodium; K, potassium; Cl, chloride; Ca, calcium; Mg, magnesium; PO_4_, phosphorus; PT, prothrombin time; APTT, activated partial thromboplastin time; INR, international normalized ratio

Laboratory investigations	Normal values (units)	Day 1	Day 2	Day 7	Day 14
Hb	11.9-15.0 (g/dL)	8.6	8.6	8.8	9.1
Hct	31-44 (%)	27.7	26.3	27	29.2
TLC	6.0-17.0 (x10^9^/L )	1.2	1.2	1.3	1.3
PLT	150-400 (x10^9^/L)	22	12	7	9
CRP	<5 (mg/dL)	49.1	57	74	40
BUN	7-20 (mg/dL)	15	13	22	20
Cr	0.2-0.5 (mg/dL)	0.1	0.1	0.1	0.3
Na	135-146 (mEq/L)	145	151	156	154
K	3.5-5.0 (mEq/L)	3.7	3.0	3.5	3.1
Cl	95-106 (mEq/L)	111	106	110	106
Ca	8.8-10.6 (mg/dL)	5.6	8.0	6.8	6.9
Mg	1.4-1.7 (mg/dL)	1.5	1.9	1.8	1.7
PO_4_	3.2-6.3 (mg/dL)	2.4	2.8	3.0	2.1
PT	10-12 (seconds)	18	10.5	10.4	9.6
APTT	22-41 (seconds)	40	26	25	25
INR	<1.1	1.2	1.3	1.3	1.1

**Figure 1:**
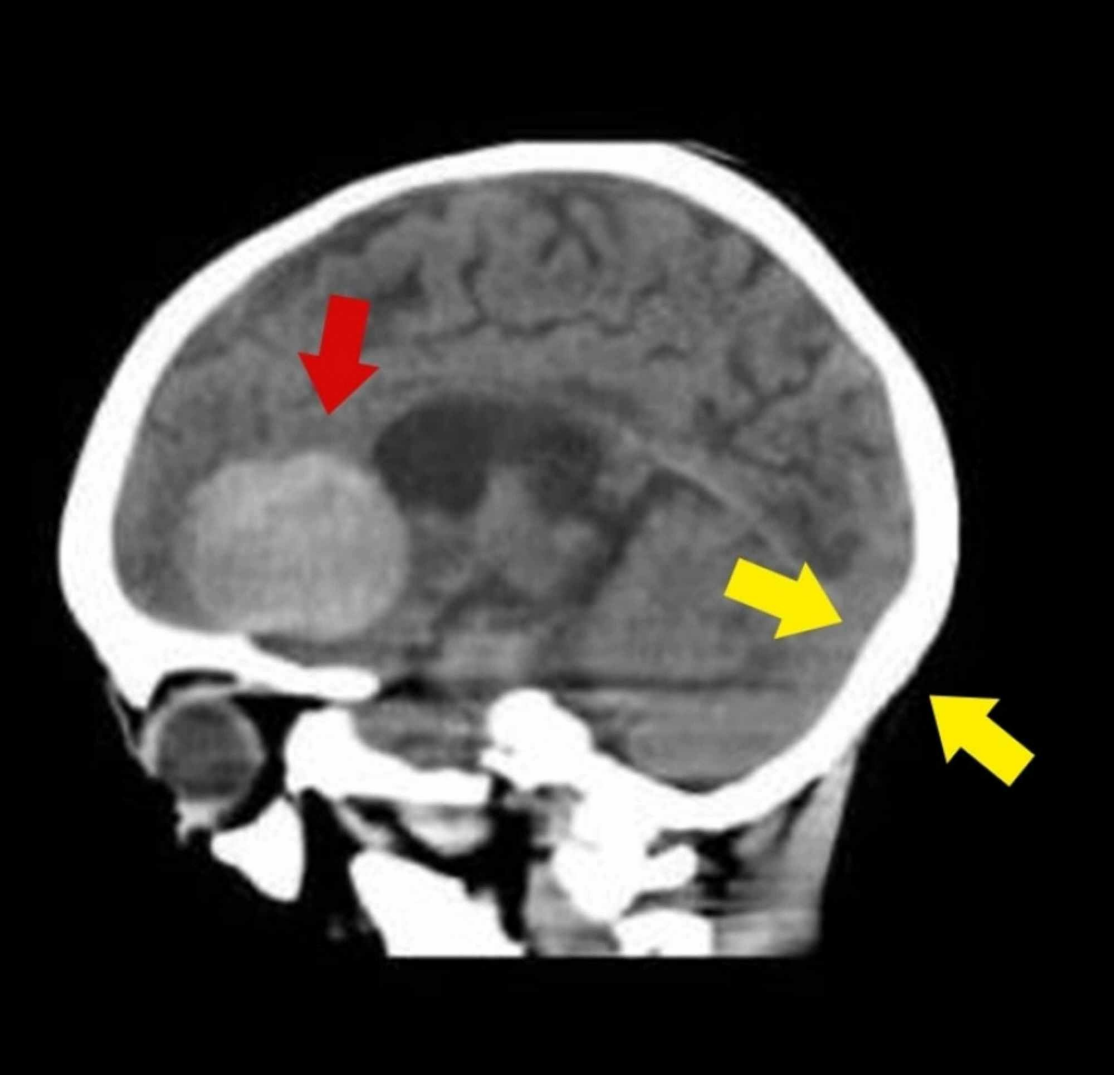
CT scan brain showing a large right frontal lobe hemorrhage (red arrow) and cortical thickening of the skull (yellow arrows).

**Figure 2:**
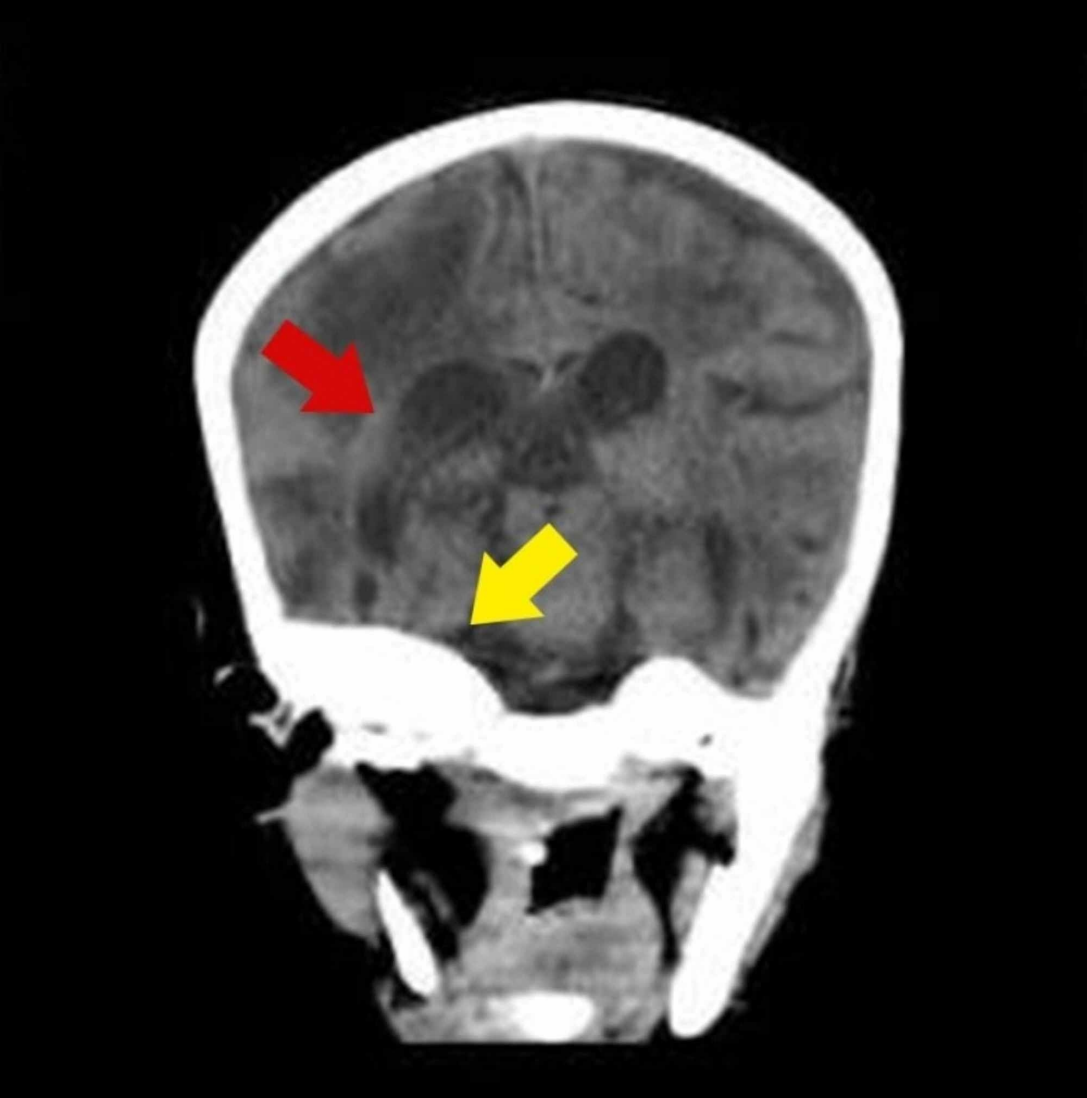
CT scan brain showing right frontal lobe lesion causing mass effect, inflammation, loss of gyri and sulci (red arrow), and cortical thickening of the skull (yellow arrow).

A CT scan was carried out, which demonstrated cortical thickening of the skull and a massive right frontal lobe hemorrhage, causing mass effect and inflammation leading to loss of gyri and sulci (Figures 1, 2). The neurosurgical consult was sought, and decompression craniectomy was advised, but surgery was not performed on account of pancytopenia and risk of bleeding. However, several measures were taken to prevent a rise in the intracranial pressure, including bed elevation, prevention of hypothermia, administration of mannitol and beta-blocker (labetalol), and the antiepileptic medications. Bone marrow biopsy was carried out, which showed erythropoiesis, myelopoiesis, and few megakaryocytes with no evidence of hemoparasites. A consult for hematopoietic stem cell transplantation (HSCT) was sought but was dismissed due to financial constraints and the development of complications. The pancytopenia worsened during the stay. A severely depleted platelet count of 7 x 10^9^/L was seen on the seventh day of admission for which multiple immediate platelet transfusions were carried out. 

The convulsions occurred almost every other day and were not controlled with the prescribed antiepileptic drugs. Levetiracetam was added to the regime on the eighth day. The child also developed an episode of hematuria on the 10th day and multiple episodes of bleeding from nose and mouth on the 13th day of admission. The GCS score of the patient declined significantly throughout the stay, and the family was counseled regarding the deteriorating condition of their child. A do-not-resuscitate (DNR) code was decided. Unfortunately, the patient expired on the 15th day of admission due to a cardiopulmonary arrest.

## Discussion

MIOP is a rare AR osteosclerotic type of skeletal dysplasia that typically presents with unique radiographic appearance of generalized hyperostosis called “bone within a bone” appearance in infants. This excessive growth of bone usually involves the medullary portion with relative sparing of the cortex [6]. The significant increase in bone density can result in the weakening of the bone, which consequently increases predisposition to osteomyelitis and fractures. It is usually diagnosed before one year of age with complaints of failure to thrive and growth retardation [1]. However, associated complications secondary to bony defects include nasal stuffiness due to paranasal or mastoid sinus malformation, cranial nerve compression due to failure of complete widening of neural foramina, deafness, proptosis, delayed dentition, mandibular osteomyelitis due to abnormal blood supply, and fragility fractures. Moreover, replacement of bone marrow by defective osseous tissue causes bone marrow failure with pancytopenia. Extramedullary hematopoiesis may also occur with resultant hepatosplenomegaly and hemolysis [1]. Our patient presented with generalized tonic-clonic seizures, nystagmus, and facial twitching secondary to frontal lobe hemorrhage and hypocalcemia.

MIOP most commonly occurs due to mutations in the TCIRG1 gene, which encodes a protein subunit called the alpha-3 subunit of the vacuolar H+‐ATPase [7]. It is involved in the phenomenon of extracellular acidification, while osteoclast resorbs the mineralized bone. Heterozygous mutations in CLCN7, which encodes Cl-/H+ exchanger, result is a less severe form of osteopetrosis and produce skeletal mineralization defects [7]. Both types of mutations result in abnormal acidification required for normal osteoclast function. Other milder form of ARO can occur due to mutations of PLEKHM1 gene, which encodes an adaptor protein essential for bone resorption and plays a significant role in vesicular transport in the osteoclasts. [8]

MIOP is typically diagnosed on classical clinical and hematological findings and characteristic radiological abnormalities of increased bone density [9]. Our child had typical osseous and dental features of MIOP, i.e., frontal bossing, flattened malar eminence, deviation of the mouth to the left side, and malformed teeth with dental caries. Rickets has also been observed as a common complication of MIOP [10]. Signs of vitamin D deficiency, such as the rachitic rosary and bowed legs, were also observed in our case. Abnormal fibrous bone tissue replaces the marrow cavity and consequently decreases hematopoietic activity. The decreased hematopoietic activity, in turn, increases extramedullary hematopoiesis, which leads to hepatosplenomegaly [1]. Furthermore, hypersplenism increases hemolysis and worsens the anemia, which causes unconjugated hyperbilirubinemia. Neutropenia predisposes to increased infections, while thrombocytopenia increases the chances of bleeding. There is a 75% chance of development of hematologic abnormalities in the first year of life. The onset of hematologic impairment within three months is predictive of adverse outcomes [9,11]. In our case, specific hematologic complications secondary to MIOP were anemia, leukopenia, and thrombocytopenia with clumped platelets for which the patient was transfused with blood multiple times. However, the coagulation profile was in the normal range. The blood culture of our patient revealed the presence of staphylococcal infection for which the patient was administered with antibiotics. Nevertheless, the condition of the child did not improve.

MIOP can also present with neurological complications. The most commonly observed neurological manifestation of MIOP occurs as a consequence of an obstruction of neural foramina through which cranial nerves, spinal cord, and major blood vessels enter or exit the skull [12]. Blindness, hearing loss, facial nerve palsy, and hydrocephalus are among the significant complications of MIOP. A rise in intracranial pressure inducing hydrocephalus is caused by increased thickness of calvarium along with a decrease in cranial capacity. This calvarial deformity and resultant raised intracranial pressure are extremely severe and require immediate surgical intervention (e.g., decompression craniectomy) [13]. Children with MIOP are also at increased risk of developing hypocalcemia with resultant tetanic seizures and secondary hyperparathyroidism [14]. Biochemical investigation in our patient also revealed hypocalcemia for which calcium gluconate diluted in 10% dextrose water was administered. Also, facial twitching and deviation of the mouth to the left side were observed. However, the cause of tonic-clonic seizure in our case could probably be frontal lobe hemorrhage with hydrocephalus. The patient was managed on phenytoin, diazepam, and sodium valproate for the alleviation of seizures. Multiple measures to decrease intracranial pressure (bed elevation, prevention of hypothermia, and administration of mannitol and labetalol) were also performed. However, no improvement was seen, and the condition of the baby deteriorated eventually despite adding levetiracetam to the regimen.

If untreated, MIOP is usually lethal by the first year of life. Death in MIOP commonly occurs due to complications secondary to anemia, bleeding, or infections. At present, the only chance of cure for MIOP is HSCT. HSCT should be performed soon before the child develops irreversible neurological impairment. Abnormal osteoclasts and precursor cells in osteopetrosis patients are replaced by normal cells. Due to a greater chance of morbidity and mortality, HSCT is usually advised for the most severe cases of osteopetrosis [15]. Osteopetrosis patients transplanted with allogeneic donor stem cells have achieved successful results with HSCT. Moreover, MIOP can also be treated with non-allogeneic HSCT which depicts higher survival rates [4]. Unfortunately, HSCT could not be performed in our case due to financial constraints and rapid development of complications, such as frontal lobe hemorrhage, tonic-clonic seizures, and multiple episodes of bleeding. However, supportive treatment with symptomatic management of complications with antiepileptics, antibiotics, and fluids was done. The child expired on the 15th day of admission due to cardiopulmonary arrest. Genetic counseling of such cases is important. Antenatal radiographs can help in early diagnosis of MIOP in the families and can help in following better options like HSCT before the development of lethal neurological complications [16].

## Conclusions

Early and prompt diagnosis based on clinical, radiographic, and genetic evidence is crucial in cases of MIOP since the disorder is associated with a reduced life expectancy, as depicted by this case. Most cases of MIOP die within the first decade of life as a result of various complications and a delayed diagnosis. No definitive medical treatment has yet been identified. A multidisciplinary approach should be adopted, along with supportive treatment and systemic management of complications. HSCT is the only available long-term cure.
